# Association between Dietary Live Microbe Intake and Periodontitis in Adults: Evidence from NHANES

**DOI:** 10.3290/j.ohpd.c_2026

**Published:** 2025-06-03

**Authors:** Shanshan Gong, Bin Lv, Yihong Fan, Yuchang Fei

**Affiliations:** a Yuchang Fei; b Department of Integrated Chinese and Western Medicine, The First People’s Hospital of Jiashan, Jiashan Hospital Affiliated of Jiaxing University, Jiaxing 314100, Zhejiang Province, China. Researched, designed, conducted the study; wrote and reviewed the manuscript.; c Shanshan Gong Department of Gastroenterology, The Third Affiliated Hospital of Zhejiang Chinese Medical University, Hangzhou 310005, Zhejiang Province, China. Researched, designed, conducted the study; wrote and reviewed the manuscript.; d Bin Lv Department of Gastroenterology, The First Affiliated Hospital of Zhejiang Chinese Medical University, Hangzhou 310006, Zhejiang Province, China. Researched, designed, conducted the study; wrote and reviewed the manuscript.; e Yihong Fan Department of Gastroenterology, The First Affiliated Hospital of Zhejiang Chinese Medical University, Hangzhou 310006, Zhejiang Province, China. Researched, designed, conducted the study; wrote and reviewed the manuscript.

**Keywords:** adult, dietary live microbe, National Health and Nutrition Examination Survey, NHANES, periodontitis

## Abstract

**Purpose:**

To investigate the relationship between the consumption of live microbes in the diet and adult periodontitis.

**Materials and Methods:**

Utilising data from the National Health and Nutrition Examination Survey (NHANES) spanning 1999–2004 and 2009–2014, 16,600 adults who underwent 24-h face-to-face dietary recall and oral health examinations were identified. Dietary live microbe intake was categorised into low, medium, and high levels. To examine the relationship between different levels of dietary live microbe intake and periodontitis, we employed logistic regression, subgroup and restricted cubic spline models.

**Results:**

Upon comprehensive covariate adjustment, low dietary live microbe intake (<104 CFU/g) demonstrated a positive association with periodontitis prevalence, while medium intake (104 to 107 CFU/g) showed a negative association. Conversely, no significant associations were observed between high dietary live microbe intake (>107 CFU/g) and periodontitis. Restricted cubic spline analysis confirmed a linear association between low dietary live microbe intake. Moreover, a U-shaped dose–response relationship was identified between medium dietary live microbe intake and periodontitis prevalence.

**Conclusions:**

Moderate intake of medium live microbe food may be more conducive to avoiding the occurrence of periodontitis.

Periodontitis is a chronic oral disease characterised by inflammation and degradation of the tissues surrounding the teeth due to dental plaque accumulation, ultimately leading to tooth mobility and loss.^43^ Notably, it stands as the primary cause of adult tooth loss.^33^ Over the past several decades, substantial evidence has demonstrated a strong association between periodontitis and various systemic diseases. These include cardiovascular disease, cancer, rheumatism, diabetes, obesity, Alzheimer’s disease, and chronic lower respiratory disease. Additionally, periodontitis increases the risk of peri-implantitis.^6,31,40,46^ All of these conditions are linked to serious health issues and mortality, as reported by the National Center for Health Statistics of the Centers for Disease Control and Prevention.^23^ The global prevalence of mild forms of periodontitis is estimated to be approximately 62% in the worldwide population, with severe forms affecting around 23.6%.^7,44^ This makes periodontitis the seventh most common disease among humans.

The human oral cavity serves as a vast micro-ecosystem hosting over 700 bacterial species, entailing dynamic interactions with the host to uphold oral micro-ecosystem equilibrium. Dysbiosis within the oral microbial community is a pivotal factor in periodontitis onset and progression.^13^ Thus, rectifying the balance by fostering beneficial bacterial predominance and regulating oral flora emerges as a critical approach to periodontitis prevention and management. While enhanced hygiene practices in food production offer public health benefits, they may inadvertently restrict microbial exposure, potentially eliciting adverse health implications. The significance of microbes is underscored by the ‘hygiene hypothesis’ positing that diminished microbial exposure incites immune dysregulation, predisposing individuals to chronic inflammatory conditions.^38^

In a recent study, Sanders and colleagues^30^ analysed the National Health and Nutrition Examination Survey (NHANES) database to investigate the presence of live microbes in various foods. They found that diets high in live microorganisms were linked to better health outcomes, including lower body mass index (BMI), blood pressure, lipid levels, glucose, and insulin levels, as well as reduced inflammation.^15^ Other studies have demonstrated that live microbes can prevent certain diseases, including postmenopausal osteoporosis, sarcopenia, and depression.^4,48,52^ Common dietary live microbes are primarily probiotics, such as *Lactobacillus*, *Bifidobacterium*, *Saccharomyces* and *Bacillus*. A network meta-analysis revealed^32^ that professional mechanical plaque removal, when combined with probiotic treatments, effectively improves probing pocket depth and clinical attachment levels in patients with periodontitis. Among the probiotics studied, *Lactobacillus* was found to be the most comprehensive and effective. Additionally, a randomised placebo-controlled clinical trial found^16^ that oral administration of *Bifidobacterium* probiotic may serve as a beneficial adjunct to scaling and root planing in chronic periodontitis. Despite these insights, the interplay between periodontitis and dietary live microbe intake remains nebulous, prompting inquiry into whether increased dietary live microbe consumption denotes superior outcomes. This study endeavours to elucidate this relationship through a cross-sectional analysis utilising the NHANES database to explore the correlation between the consumption of live microbes in the diet and adult periodontitis.

## MATERIALS AND METHODS

### Data Sources

NHANES is a research programme aimed at evaluating the health and nutritional status of adults and children in the United States. The NHANES interview covers a range of topics, including demographics, socioeconomic status, dietary habits, and health-related inquiries. Physical examinations encompassed physiological measurements, laboratory tests, and more. Utilising a stratified multi-stage sampling design, NHANES achieved a representative sample of US residents. For detailed information, please visit the NHANES website here. The NHANES protocols were approved by the institutional review boards of the National Center for Health Statistics (NCHS) (NHANES 2005–2006 NCHS IRB: Protocol #2005–06), and informed consent was obtained from all participants.

**Fig 1:**
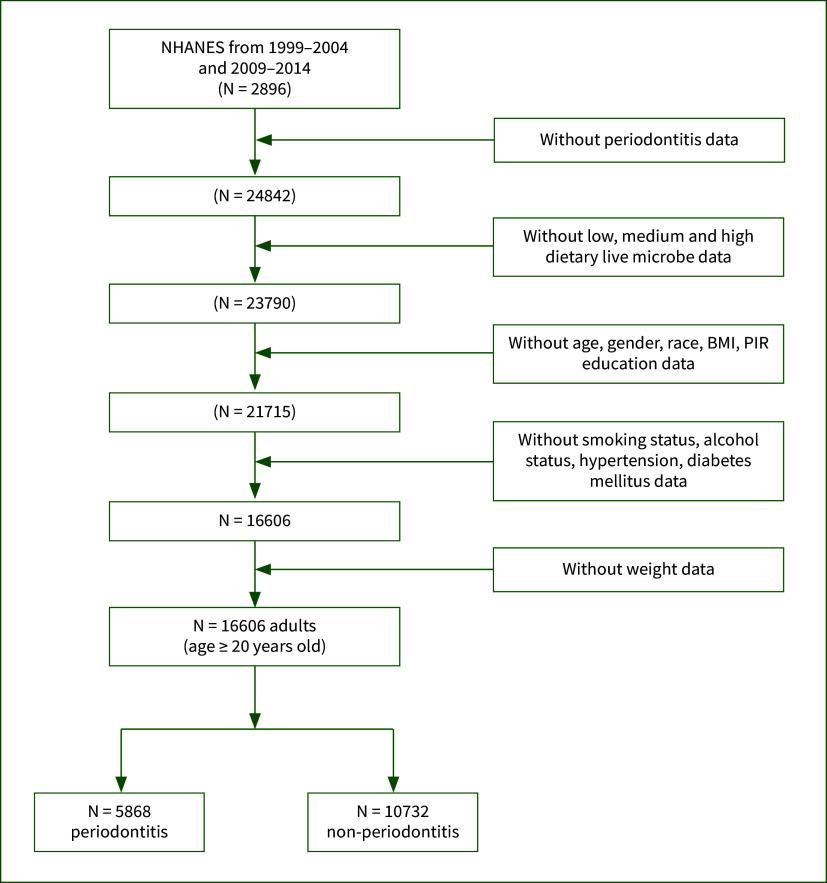
Flow chart of the study. BMI, body mass index; PIR, poverty income ratio.

We included participants from six NHANES research cycles (1999–2000, 2001–2002, 2003–2004, 2009–2010, 2011–2012, 2013–2014). Exclusion criteria were applied to patients who were: (1) under the age of 20; (2) lacking data on dietary live microbe intake or periodontitis; (3) missing data on age, gender, race, BMI, family poverty income ratio (PIR), education, smoking status, alcohol status, hypertension, diabetes mellitus (DM), or weight. After applying these exclusions, a total of 16,600 participants were included in the subsequent analyses. The selection process is outlined in Figure 1.

### Dietary Live Microbe Intake Category

The 24-hour dietary recall data from NHANES were linked by the NCHS to the US Department of Agriculture Food Surveys Nutrient Database to estimate energy and nutrient intake. A classification system developed by Sanders^30^ was employed to determine the quantity of live microbes in 9388 food codes across 48 subgroups in the NHANES database. Foods were categorised based on live microbe content per gram into low (Lo category, <10^4^ CFU/g), medium (Med category, 10^4^–10^7^ CFU/g), and high (Hi category, >10^7^ CFU/g) levels: Lo category mainly included pasteurised foods, Med category primarily consisted of unpeeled fresh fruits and vegetables, and Hi category encompassed unpasteurised fermented foods and probiotic supplements.

### Definition of Periodontitis

Periodontitis diagnosis relied on measuring periodontal pocket probing depth (PD) and attachment loss (AL). The diagnostic criteria followed the 2012 Centers for Disease Control/American Academy of Periodontology (CDC/AAP) Classification Criteria^9^ (Table 1). In this study, participants were divided into two groups: non-periodontitis and periodontitis. Subjects diagnosed with mild, moderate, or severe periodontitis were categorised into the periodontitis group.

**Table 1:** Diagnostic criteria of periodontitis

Case	Definition
No periodontitis	No evidence of mild, moderate, or severe periodontitis
Mild periodontitis	≥ 2 interproximal sites with AL ≥ 3 mm, and ≥ 2 interproximal sites with PD ≥ 4 mm (not on the same tooth) or one site with PD ≥ 5 mm
Moderate periodontitis	≥ 2 interproximal sites with AL ≥ 4 mm (not on same tooth), or ≥ 2 interproximal sites with PD ≥ 5 mm (not on same tooth)
Severe periodontitis	≥ 2 interproximal sites with AL ≥ 6 mm (not on same tooth) and ≥ 1 interproximal site with PD ≥ 5 mm
AL, attachment loss; PD, probing depth.

### Covariates

Demographic data on age, gender, race, BMI, PIR, and education were collected through surveys, while information on smoking and alcohol status was obtained through questionnaires. Diagnostic criteria for DM included clinical diagnosis, glycohemoglobin concentration ≥ 6.5%, fasting blood glucose concentration ≥ 7.0 mmol/L, plasma glucose level ≥ 11.1 mmol/L on a random or 2-hour oral glucose tolerance test, or documented use of antidiabetic medications/insulin therapy. Diagnostic criteria for hypertension comprised a clinical diagnosis by a physician, use of antihypertensive medication, or systolic blood pressure ≥ 140 mmHg or diastolic blood pressure ≥ 90 mmHg. Age categories were defined as ‘20–40 yr’, ‘41–60 yr’ and ‘>60 yr’. Race categories were ‘White’, ‘Black’, ‘Mexican American’, and ‘Other’. PIR categories were ‘<1’, ‘1–3’, and ‘≥ 3’. Education categories were ‘below high school’, ‘high school’, and ‘college or above’. BMI categories were ‘no obesity (BMI <30)’ and ‘obesity (BMI ≥ 30)’. Smoking status was categorised as ‘former’, ‘never’, and ‘current’. Alcohol status was classified as ‘former’, ‘never’, ‘mild’, ‘moderate’, and ‘heavy’.

### Statistical Analysis

All analyses accounted for the complex, multi-stage probability sampling design of NHANES by incorporating appropriate sampling weights. Continuous variables were presented as weighted means ± standard errors (SE), and categorical variables as numbers (weighted percentage). Disparities in categorical data were assessed using the Chi-square test, while differences in continuous variables were evaluated with the *t*-test (for normally distributed variables) or the Mann–Whitney test (for skewed distributions). Participants were grouped into Lo, Med, and Hi categories of dietary live microbe intake. Weighted logistic regression was used to explore the association between different levels of dietary live microbe intake and periodontitis. Three models were constructed: Crude model (unadjusted); Model 1 (adjusted for age, gender, race, PIR, education, BMI, and research cycles); Model 2 (further adjusted for smoking status, alcohol status, DM, and hypertension from Model 1). Subgroup analyses were stratified by various factors, with interaction terms employed to explore subgroup differences. Additionally, restricted cubic spline (RCS) analysis was utilised to investigate the dose–response relationship between different levels of dietary live microbe intake and periodontitis. Statistical analyses were conducted using R software (version 4.3.2), with a significance level set at P < 0.05.

## RESULTS

### Participant Characteristics

Table 2 displays the characteristics of the study population. Among the 16,600 participants, 5,868 were diagnosed with periodontitis. The weighted mean age of the participants was 47.35 ± 0.23 years, with a gender distribution of 49.32% female and 50.68% male. Participants with periodontitis were more likely to be female, have a PIR ≥ 3, not be obese, have a college education or higher, be non-smokers, engage in mild alcohol consumption, and have hypertension and DM compared to those without periodontitis.

**Table 2:** Baseline characteristics of the study participants grouped by periodontitis status

Variables	Overall	Non- periodontitis	Periodontitis	P value
(N = 16,600)	(N = 10,732)	(N = 5868)
Age, years (mean ± se)	47.35 ± 0.23	44.15 ± 0.25	55.36 ± 0.27	<0.0001
**Gender, n (%)**				
Female	8042 (49.32)	5722 (52.77)	2320 (40.70)	<0.0001
Male	8558 (50.68)	5010 (47.23)	3548 (59.30)	
**Race, n (%)**				<0.0001
Non-Hispanic White	7980 (71.98)	5516 (74.56)	2464 (65.51)	
Non-Hispanic Black	3248 (10.16)	1893 (8.94)	1355 (13.22)	
Mexican American	3036 (7.43)	1972 (6.84)	1064 (8.91)	
Other race	2336 (10.43)	1351 (9.65)	985 (12.37)	
**PIR, n (%)**				<0.0001
PIR <1	2908 (11.89)	1608 (10.35)	1300 (15.74)	
1<=PIR <3	6631 (33.71)	4025 (31.47)	2606 (39.32)	
PIR >=3	7061 (54.40)	5099 (58.18)	1962 (44.94)	
**BMI**				<0.001
No obesity	10654 (65.80)	7007 (67.08)	3647 (62.58)	
Obesity	5946 (34.20)	3725 ( 32.92)	2221 (37.42)	
**Education**				<0.0001
Below high school	4022 (14.88)	2151 (11.88)	1871 (22.40)	
High school	3783 (23.08)	2369 (22.21)	1414 (25.28)	
College or above	8795 (62.03)	6212 (65.91)	2583 (52.33)	
**Dietary live microbe intake**
**Lo category**				<0.0001
Q1	4150 (21.23)	2962 (23.55)	1188 (15.42)	
Q2	4150 (24.96)	2761 (26.26)	1389 (21.73)	
Q3	4150 (26.30)	2580 (25.56)	1570 (28.15)	
Q4	4150 (27.51)	2429 (24.64)	1721 (34.70)	
**Med category**				<0.0001
Q1	6402 (36.45)	3890 (34.92)	2512 (40.27)	
Q2	1898 (10.92)	1277 (11.12)	621 (10.41)	
Q3	4150 (25.54)	2815 (26.32)	1335 (23.58)	
Q4	4150 (27.10)	2750 (27.64)	1400 (25.74)	
**Hi category**				<0.0001
Q1	12964 (74.63)	8212 (73.59)	4752 (77.25)	
Q2	3636 (25.37)	2520 (26.41)	1116 (22.75)	
**Smoking status**				<0.0001
Never	8949 (53.45)	6350 (57.90)	2599 (42.31)	
Former	4203 (25.38)	2426 (23.43)	1777 (30.25)	
Now	3448 (21.17)	1956 (18.67)	1492 (27.43)	
**Alcohol status**				<0.0001
Never	2159 (10.90)	1409 (11.32)	750 (9.87)	
Former	2856 (14.19)	1551 (12.08)	1305 (19.47)	
Mild	5821 (37.20)	3859 (37.83)	1962 (35.61)	
Moderate	2521 (17.64)	1810 (18.79)	711 (14.76)	
Heavy	3243 (20.07)	2103 (19.99)	1140 (20.29)	
**Hypertension**				<0.0001
No	9811 (63.48)	7046 (68.47)	2765 (50.98)	
Yes	6789 (36.52)	3686 (31.53)	3103 (49.02)	
**DM**				<0.0001
No	14102 (89.13)	9597 (92.24)	4505 (81.34)	
Yes	2498 (10.87)	1135 (7.76)	1363 (18.66)	
**Research cycles**				<0.0001
1999–2000	2308 (14.61)	1885 (17.68)	423 (6.92)	
2001–2002	2909 (18.25)	2434 (22.43)	475 (7.78)	
2003–2004	2672 (17.35)	2285 (21.76)	387 (6.31)	
2009–2010	3079 (15.60)	1319 (10.96)	1760 (27.20)	
2011–2012	2646 (16.69)	1227 (12.63)	1419 (26.84)	
2013–2014	2986 (17.50)	1582 (14.53)	1404 (24.95)	
Lo, low dietary live microbe intake; Med, medium dietary live microbe intake; DM, diabetes mellitus; BMI, body mass index; PIR, poverty income ratio.

### Weighted Multivariate Logistic Regression Analysis for the Association Between Different Levels of Dietary Live Microbes Intake and Periodontitis

**Fig 2:**
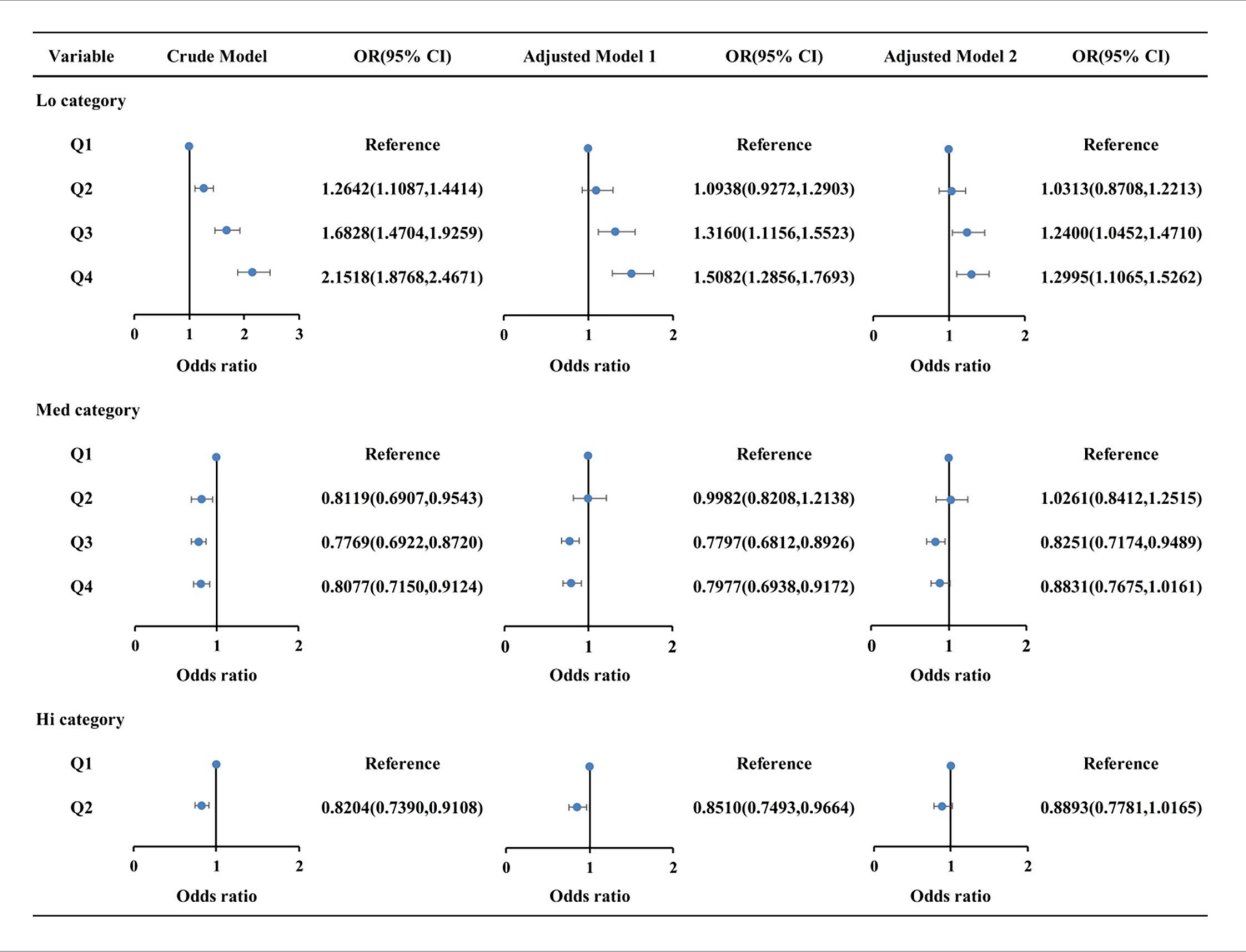
Associations between different levels of dietary live microbe intake and periodontitis.Crude model: No adjustment for any potential influence factors. Model 1: Adjusted for age, gender, race, PIR, education, BMI and research cycles. Model 2: Further adjustment for smoking status, alcohol status, DM, and hypertension. Lo, low dietary live microbe intake; Med, medium dietary live microbe intake; Hi, high dietary live microbe intake; OR, odds ratio; CI, confidence interval; DM, diabetes mellitus; BMI, body mass index; PIR, poverty income ratio.

Figure 2 illustrates the results of the weighted multivariate logistic regression analysis investigating the relationship between different levels of dietary live microbe intake and periodontitis. The Lo category (Q1 [33, 1810.085 g/d], Q2 (1810.085, 2555.24 g/d], Q3 (2555.24, 3509.847 g/d], and Q4 (3509.847, 18069.95 g/d]) and Med category (Q1[0 g/d], Q2 [0, 39.71 g/d], Q3 [39.71, 153.812 g/d], and Q4 [153.812, 1680 g/d]) were divided into four groups based on dosage, while the Hi category (Q1 [0 g/d], Q2 [0, 918.76 g/d]) was separated into two groups.

In the crude model, the Lo category showed an increased prevalence of periodontitis, while the Med and Hi categories were associated with a decreased prevalence. After adjusting for age, gender, race, education, PIR, BMI, and research cycles (Model 1), the prevalence of periodontitis was significantly higher in the Q3 and Q4 groups of the Lo category and lower in the Q3 and Q4 groups of the Med category, as well as the Q2 group of the Hi category (P < 0.05). Further adjustments in Model 2 (adding smoking, alcohol status, DM and hypertension) revealed significantly higher prevalence of periodontitis in the Q3 and Q4 groups of the Lo category and a lower prevalence in the Q3 group of the Med category (P < 0.001). Notably, no significant association with periodontitis was found in the Hi category.

### Restricted Cubic Spline (RCS)

**Fig 3a and b:**
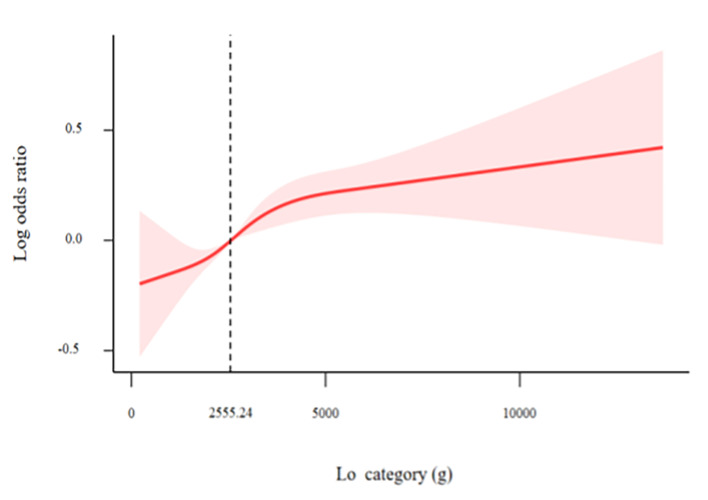

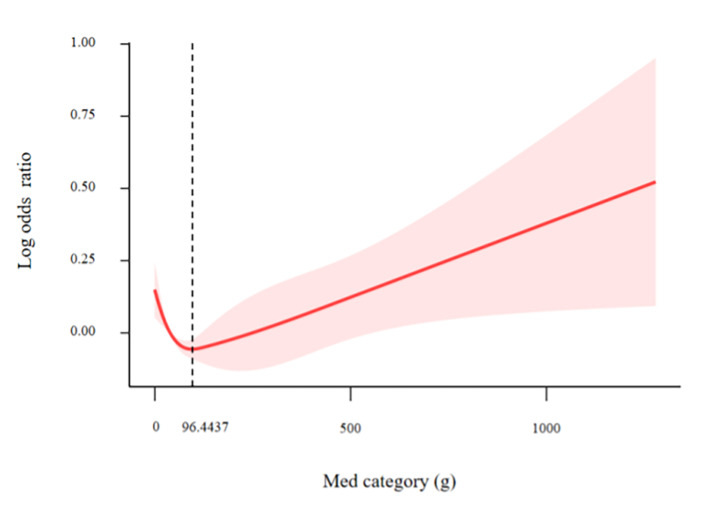
Dose–response relationships between Lo (a), Med (b) categories and periodontitis. (a) Lo category, (b) Med category. The model was adjusted for age, gender, race, PIR, education, BMI, research cycles, smoking status, alcohol status, DM, and hypertension. DM, diabetes mellitus; BMI, body mass index; PIR, poverty income ratio.

An investigation into the exposure-response relationship between the Lo and Med categories and periodontitis was conducted using RCS. The exposure-response curve for the Lo category and periodontitis displayed an increasing trend (P for nonlinear = 0.0787, P for overall association < 0.001) (Fig 3a). Conversely, the curve for the Med category (P for nonlinear < 0.001, P for overall association < 0.001) (Fig 3b) showed a U-curve trend, indicating a negative association with periodontitis below 96.4437 g and a positive association above this threshold.

### Subgroup Analyses

Subgroup analyses, interaction tests, and trend assessments were performed across various demographics and health factors to investigate the association between the Lo and Med categories and periodontitis. Table 3 and Table 4 present all subgroup analysis outcomes in the Lo and Med categories. In the Lo category, females, white individuals, those with a PIR of ≥ 3, below high school education, history of alcohol use, and current heavy drinkers exhibited a higher prevalence of periodontitis. Only the interaction test for research cycles yielded significant results. Conversely, in the Med category, individuals aged 20–40 years, with a PIR between 1 and 3, below high school education, non-obese, current non-smokers, and without DM or hypertension showed a lower prevalence of periodontitis. No significant interactions were observed in the Med category.

**Table 3:** Subgroup analyses and interaction effects on the association between Lo category with periodontitis

Duration/Subgroup	Q1	Q2	Q3	Q4	P for trend	P for interaction
aOR [95%CI]
**Age**						0.157
20–40 yr	reference	1.162 (0.772, 1.751)	1.512 (1.093, 2.092)	1.297 (0.891, 1.886)	0.226	
41–60 yr	reference	0.860 (0.646, 1.144)	0.994 (0.766, 1.290)	1.098 (0.851, 1.415)	0.121	
>60 yr	reference	1.076 (0.849, 1.364)	1.145 (0.883, 1.485)	1.043 (0.758, 1.437)	0.68	
**Gender**	reference					0.22
Female	reference	0.961 (0.781, 1.182)	1.215 (0.975, 1.515)	1.439 (1.115, 1.856)	<0.001	
Male	reference	1.164 (0.890, 1.522)	1.281 (0.970, 1.691)	1.249 (0.962, 1.621)	0.137	
**Race**	reference					0.619
White	reference	1.038 (0.828, 1.301)	1.278 (0.998, 1.638)	1.397 (1.109, 1.760)	<0.001	
Black	reference	1.025 (0.758, 1.387)	1.069 (0.758, 1.507)	0.845 (0.568, 1.256)	0.5	
Mexican American	reference	0.807 (0.571, 1.141)	1.048 (0.654, 1.681)	0.947 (0.675, 1.328)	0.733	
Other	reference	1.103 (0.706, 1.722)	1.271 (0.850, 1.899)	1.222 (0.797, 1.874)	0.275	
**PIR**	reference					0.503
PIR <1	reference	0.773 (0.521, 1.148)	0.792 (0.527, 1.191)	0.954 (0.645, 1.409)	0.926	
1<=PIR <3	reference	0.953 (0.748, 1.214)	1.191 (0.877, 1.616)	1.275 (0.934, 1.740)	0.062	
PIR >=3	reference	1.210 (0.933, 1.569)	1.477 (1.155, 1.887)	1.483 (1.149, 1.915)	0.002	
**Education**	reference					0.4
Below high school	reference	0.932 (0.673, 1.290)	1.133 (0.812, 1.582)	1.353 (1.022, 1.792)	0.01	
High school	reference	1.116 (0.777, 1.603)	1.081 (0.754, 1.550)	1.310 (0.913, 1.880)	0.15	
College or above	reference	1.021 (0.814, 1.281)	1.320 (1.045, 1.667)	1.287 (1.021, 1.622)	0.006	
**BMI**	reference					0.764
No obesity	reference	1.016 (0.829, 1.245)	1.224 (0.980, 1.528)	1.363 (1.106, 1.679)	<0.001	
Obesity	reference	1.090 (0.807, 1.474)	1.336 (0.984, 1.814)	1.261 (0.915, 1.738)	0.122	
**Smoking status**	reference					0.3506
Former	reference	0.9895 (0.7336, 1.3346)	1.3626 (1.0390, 1.7871)	1.2375 (0.9147, 1.6741)	0.0438	
Never	reference	1.0019 (0.8051, 1.2469)	1.0533 (0.8417, 1.3181)	1.2562 (0.9991, 1.5795)	0.0307	
Now	reference	1.1089 (0.7531, 1.6327)	1.4828 (0.9958, 2.2080)	1.2976 (0.9370, 1.7971)	0.0804	
**Alcohol status**	reference					0.2907
Moderate	reference	1.1521 (0.7290, 1.8207)	1.2957 (0.7995, 2.0999)	1.2269 (0.7165, 2.1009)	0.5027	
Never	reference	1.5646 (1.0182, 2.4042)	1.0654 (0.6820, 1.6641)	1.6618 (1.0492, 2.6323)	0.1131	
Mild	reference	1.0798 (0.8429, 1.3833)	1.2027 (0.9092, 1.5910)	1.2220 (0.9368, 1.5942)	0.1433	
Heavy	reference	0.6987 (0.4415, 1.1056)	1.2592 (0.8101, 1.9573)	1.1536 (0.7798, 1.7064)	0.0448	
Former	reference	0.8104 (0.5798, 1.1328)	1.1904 (0.8283, 1.7107)	1.3997 (0.9247, 2.1188)	0.0255	
**DM**	reference					0.804
No	reference	1.0235 (0.8556, 1.2244)	1.2526 (1.0371, 1.5130)	1.3407 (1.1331, 1.5862)	<0.001	
Yes	reference	1.0711 (0.7270, 1.5780)	1.2049 (0.8214, 1.7674)	1.0105 (0.6790, 1.5039)	0.884	
**Hypertension**	reference					0.267
Yes	reference	1.1174 (0.8678, 1.4388)	1.1701 (0.9019, 1.5179)	1.1353 (0.8913, 1.4460)	0.3315	
No	reference	0.9315 (0.7544, 1.1502)	1.2545 (0.9905, 1.5889)	1.3956 (1.1182, 1.7420)	<0.001	
**Research cycles**	reference					0.0042
1999–2000	reference	1.2048 (0.8997, 1.6134)	1.5192 (1.0406, 2.2179)	1.0138 (0.5047, 2.0366)	0.4381	
2001–2002	reference	1.3469 (0.8302, 2.1851)	1.2491 (0.6660, 2.3430)	1.6686 (1.0468, 2.6597)	0.0989	
2003–2004	reference	1.4822 (0.8551, 2.5691)	1.5384 (0.9159, 2.5839)	1.9072 (1.1719, 3.1040)	0.0041	
2009–2010	reference	0.9997 (0.6904, 1.4474)	1.3019 (0.9201, 1.8422)	1.1290 (0.7372, 1.7291)	0.4698	
2011–2012	reference	0.4672 (0.3287, 0.6642)	0.5762 (0.3928, 0.8452)	0.8653 (0.5900, 1.2691)	0.113	
2013–2014	reference	1.0700 (0.6836, 1.6751)	1.4435 (0.8909, 2.3390)	1.1850 (0.8243, 1.7034)	0.1892	
Each subgroup analysis was adjusted for age, gender, race, PIR, education, BMI, research cycles, smoking status, alcohol status, DM, and hypertension, except for the subgroup variable. Lo, low dietary live microbe intake; OR, odds ratio; CI, confidence interval; DM, diabetes mellitus; BMI, body mass index; PIR, poverty income ratio.

## DISCUSSION

Periodontitis is notably characterised by dysbiosis of the microbial community. Diet plays a crucial role in the typical oral dysbiosis of periodontitis as it provides nutrients for microorganisms, creates microenvironments conducive to the colonisation and survival of periodontal pathogenic bacteria, and can inhibit the growth of other microorganisms. With the evolution of modern society, changes in eating habits and processing technologies have led to a decrease in the intake of dietary live microbe compared to our ancestors.

Sanders30 developed a classification system to estimate the number of live microbes in various food codes within the NHANES database. Foods were categorised into Lo category, Med category, and Hi category based on the dietary live microbe content per gram. Previous research has shown that Lo category is linked to an increased risk of osteoarthritis^12^ and stroke,^14^ while Med category is associated with enhanced cognitive function^29^ and cardiovascular health.^47^ On the other hand, Hi category is inversely related to chronic constipation^52^ and depressive symptoms.^48^ However, the relationship between different levels of dietary live microbe intake and periodontitis remains unclear. Based on an analysis of six NHANES data cycles, it was found that Lo category was associated with an elevated risk of periodontitis. Surprisingly, the Med category, rather than the Hi category, was linked to a reduced incidence of periodontitis.

Dietary live microbe, abundant in various probiotics, particularly the Med and Hi categories, have the ability to directly interact with the existing oral microbiota. These probiotics can competitively exclude or inhibit pathogenic bacteria linked to periodontitis, fostering a healthier microbial balance in the oral cavity. The use of probiotics in addressing periodontal disease predominantly focuses on lactobacillus strains, showcasing differences in the oral *Lactobacillus* composition between individuals with periodontitis and those who are healthy. The most prevalent probiotic strains in the saliva of healthy individuals include *Lactobacillus griseus* and *Lactobacillus fermentum*, while *Lactobacillus plantarum* is predominant in periodontitis patients.^8^ Probiotics aid in reconstituting the oral microbiota by inhibiting pathogenic bacteria.^34^ Koll-Klais et al^24^ found that *Lactobacillus* detected *in vivo* had a certain inhibitory effect on periodontal pathogens and cariosis-associated *Streptococcus*, with strain differences. Studies have shown that certain probiotics can disrupt the cell walls of pathogenic bacteria, secrete anti-microbial compounds, and lower biofilm pH.^39^ *In vitro* experiments by Radaic et al^36^ found that *Lactococcus lactis *and its anti-microbial peptide Nisin effectively inhibited the growth of biofilms containing periodontal pathogenic bacteria. Additionally, oral administration of *Lactobacillus* has been found to significantly reduce the levels of five key periodontal pathogens, including *Porphyromonas gingivalis* and *Aggregatibacter actinomycetemcomitans*.

Probiotics have shown efficacy in the clinical treatment of patients with periodontitis. A study by Alshareef et al^1^ revealed that utilising probiotic lozenges containing *Lactobacillus acidophilus*, *Lactobacillus casei*, and rhamnose saliva *Lactobacillus* led to a significant decrease in the bleeding index compared to standard periodontal treatment. Patients undergoing *Lactobacillus Royale*-assisted periodontal therapy exhibited significantly lower counts of *Porphyromonas gingivalis* and experienced reduced periodontal pocket depths.^20^ Furthermore, oral administration of recombinant *Lactobacillus brevis* tablets resulted in a notable reduction in deep periodontal pockets and mean pocket depths, particularly in patients with moderate to deep pockets.^26^ Research has indicated that cultures of *Streptococcus salivarius* K12 and M18 exhibited inhibitory effects on key periodontal pathogens,^19^ while toothpaste containing *Lactobacillus* and *Bifidobacterium* helped reduce levels of certain periodontal pathogens.^5^

Among the 9388 food codes in the NHANES database, nearly 8954 were classified as Lo category, including heat-treated processed foods and undercooked mixed salads, with meat dominating this category. Overconsumption of meat, as a primary source of animal protein, may elevate the risk of diseases such as hypertension, DM, and hyperlipidemia. DM has been identified as a contributing factor to the development of periodontitis, with it being listed as the sixth most common complication of DM.^28^ Studies have shown that diabetic patients have an increased risk of periodontitis compared to non-diabetic individuals, with poorly controlled DM significantly raising the risk of periodontitis.^21,50^ Mechanisms through which diabetes exacerbates periodontitis include alterations in oral flora, inflammatory factors, adipokine levels, and oxidative stress.^35^ Systemic inflammation associated with hypertension can trigger local periodontal inflammatory responses, leading to tissue destruction and tooth loss. In addition, hypertension may also lead to arteriolar dysfunction, affecting local nutrient supply and aggravating periodontal inflammation.^51^ Hyperlipidaemia may enhance the risk of periodontitis by compromising the host immune response and affecting bone metabolism, leading to inflammatory disturbances in periodontal tissues.^22,27^

Micronutrients like vitamins A, B, C, D, and E are predominantly found in the Med or Hi categories, and their deficiency has been linked to periodontal disease.^45^ Vitamin A, a fat-soluble vitamin, plays a crucial role in maintaining epithelial cell integrity and exhibits potential antioxidant properties, making it a valuable component in both non-surgical and surgical periodontal therapies.^2,3^ Vitamin B_12_, primarily sourced from meat, is associated with an increased risk of periodontal disease when levels are low.^54^ Vitamin C shows promise in promoting periodontal health, with two clinical studies confirming that higher fruit intake rich in vitamin C can reduce gum and periodontal inflammation.^42,49^ Vitamin D, essential for immune regulation, exerts broad anti-inflammatory effects.^10,17^ Research by Laky et al^25^ identified a higher prevalence of vitamin D deficiency in periodontal disease patients compared to healthy individuals. Moreover, studies have shown a negative association between vitamin D levels and clinical attachment loss as well as tooth loss.^41^ Patients with periodontal disease exhibit lower levels of vitamin E compared to their healthy counterparts. In a prospective study spanning two years, Iwasaki et al^18^ found that increased vitamin E intake was inversely related to periodontal disease progression.

The RCS curve revealed a U-shaped correlation between the Med category and periodontitis. Intake levels less than 96.4437 g in the Med category were significantly associated with reduced risk of periodontitis, whereas intake levels exceeding 96.4437 g showed a marked increase in risk. Optimal consumption of foods classified under the Med category may be more favourable in preventing the onset of periodontitis. However, it is crucial to acknowledge that the estimation of 96.4437 g in this study serves as a preliminary observation and does not offer direct dietary advice. Further research is warranted to establish specific nutritional guidelines.

Interestingly, our study uncovered a lower incidence of periodontitis associated with the Med category rather than the Hi category. This outcome suggests that a higher intake of dietary live microbe may not necessarily yield superior results. The presence of various microorganisms beyond probiotics within dietary live microbes, coupled with microbial strain differences and inter-individual variability in gut microbiota, may contribute to diverse outcomes. Furthermore, it is essential to recognise that factors beyond dietary live microbes within food may also influence research findings.

Previously, a study by Lin et al^11^ explored the connection between dietary live microbe and periodontitis. However, their study concentrated on the relationship between a combined category of Medium and High (MedHi) foods and periodontitis, leaving the association with Lo, Med, and Hi categories unclear. Lin et al noted that the MedHi category was independently linked to a reduced risk of periodontitis. To validate this, we further elucidated the link between the MedHi category and periodontitis incidence (Supplementary Material). Upon adjusting for all variables, we observed significantly lower periodontitis prevalence in the Q3 group compared to Q1 within the MedHi category. Employing RCS analysis, a U-shaped trend (P for nonlinear <0.001, P for overall association <0.001) was discerned in the association between the MedHi category and periodontitis, indicating a negative correlation below 218.1941 g intake and a positive correlation beyond this threshold. These results affirm our previous conclusion that higher dietary live microbe intake does not equate to better outcomes, potentially influenced by our extended study duration, larger sample size, and adjusted covariates.

Dietary intake of live microbes poses fewer risks and boasts fewer adverse effects than traditional drug therapy. Despite the potential advantages of heightened intake, caution must be exercised, especially for individuals with cancer, autoimmune disorders, transplants, the elderly, and those with intestinal complications or severe infections. Probiotics, although quite rare, have been reported to induce sepsis in isolated cases.^37^ Furthermore, the efficacy of dietary interventions rich in live microbes may vary based on the microbial strains present, necessitating personalised approaches tailored to individual health objectives.

This groundbreaking study delves into the correlation between different levels of dietary live microbe intake and periodontitis prevalence in US adults. Leveraging data from the nationally representative NHANES database with stringent quality control measures, we sought to ensure the effectiveness of our analysis. Nevertheless, several limitations persist in this study. Firstly, the use of 24-hour dietary recall data may be subject to inaccuracies stemming from recall bias. Secondly, employing Sanders’ classification system for live dietary microbes, while efficient, may pose inaccuracies compared to direct microbial content measurements due to associated costs and time constraints. The study’s basic categorisation into Lo, Med, and Hi categories without precise quantification may introduce errors, necessitating further investigation for accurate assessment of daily dietary live microbes. Additionally, the study assumes an association without determining causality, applies solely to the American population, and acknowledges the potential presence of unaccounted confounding variables.

## CONCLUSIONS

Lo category was positively associated with periodontitis, Med category was negatively associated with periodontitis, and Hi category was not associated with periodontitis. Individuals should be advised to reduce their dietary intake of Lo live microbes. Moderate intake of Med category food may be more conducive to avoiding the occurrence of periodontitis. More intake of dietary with Hi category is not necessarily better.

### Acknowledgements

The NHANES study, conducted by the NCHS, provided the data used in this paper. The NHANES participants and researchers are acknowledged for their contributions to this significant study by the authors.

#### Funding

This research was funded by the Basic Public Welfare Research Programme of Zhejiang Province (grant number LQ23H270008) and Zhejiang Traditional Chinese Medicine Administration (grant number 2023ZL464).

#### Availability of data and materials

Publicly available data sets were analysed in this study. Data for this study are available at https://www.cdc.gov/nchs/nhanes/index.htm.

#### Declarations

##### Ethics approval and consent to participate

NHANES is conducted by the Centers for Disease Control and Prevention (CDC) and the National Center for Health Statistics (NCHS). And the NHANES study protocol was reviewed and approved by the NCHS Research Ethics Review Committee. All participants in NHANES provided written informed consent.

##### Consent for publication

Not applicable.

##### Competing interests

The authors declare no competing interests.

**Table 4:** Subgroup analyses and interaction effects on the association between Med category with periodontitis

Duration/Subgroup	Q1	Q2	Q3	Q4	P for trend	P for interaction
aOR [95%CI]
**Age**						0.068
20–40 yr	reference	1.088 (0.747, 1.583)	0.748 (0.557, 1.006)	0.662 (0.496, 0.885)	0.002	
41–60 yr	reference	1.078 (0.838, 1.386)	0.837 (0.692, 1.013)	0.983 (0.806, 1.198)	0.445	
>60 yr	reference	0.901 (0.644, 1.260)	1.008 (0.788, 1.290)	0.979 (0.774, 1.238)	0.963	
**Gender**	reference					0.612
Female	reference	1.049 (0.795, 1.386)	0.766 (0.607, 0.967)	0.894 (0.746, 1.073)	0.055	
Male	reference	1.015 (0.763, 1.350)	0.889 (0.719, 1.099)	0.875 (0.727, 1.054)	0.119	
**Race**	reference					0.976
White	reference	1.019 (0.792, 1.312)	0.830 (0.689, 1.000)	0.893 (0.736, 1.083)	0.119	
Black	reference	1.096 (0.753, 1.597)	0.762 (0.570, 1.017)	0.784 (0.541, 1.137)	0.074	
Mexican American	reference	0.897 (0.558, 1.443)	1.092 (0.741, 1.608)	0.990 (0.687, 1.427)	0.872	
Other	reference	1.036 (0.667, 1.609)	0.774 (0.544, 1.102)	0.836 (0.605, 1.155)	0.179	
**PIR**	reference					0.158
PIR <1	reference	0.918 (0.564, 1.494)	0.872 (0.628, 1.210)	0.875 (0.615, 1.246)	0.366	
1 <=PIR <3	reference	1.230 (0.910, 1.662)	0.791 (0.622, 1.005)	0.723 (0.563, 0.929)	0.004	
PIR >=3	reference	0.903 (0.659, 1.236)	0.824 (0.674, 1.009)	0.958 (0.771, 1.191)	0.528	
**Education**	reference					0.386
Below high school	reference	1.116 (0.757, 1.644)	0.625 (0.473, 0.826)	0.680 (0.514, 0.900)	<0.001	
High school	reference	1.048 (0.698, 1.573)	0.906 (0.675, 1.215)	0.937 (0.697, 1.259)	0.539	
College or above	reference	0.980 (0.729, 1.318)	0.867 (0.715, 1.053)	0.921 (0.771, 1.099)	0.253	
**BMI**	reference					0.237
No obesity	reference	1.028 (0.788, 1.342)	0.758 (0.640, 0.899)	0.768 (0.644, 0.916)	<0.001	
Obesity	reference	1.017 (0.737, 1.403)	0.923 (0.747, 1.141)	1.084 (0.851, 1.382)	0.732	
**Smoking status**	reference					0.7755
Former	reference	0.8470 (0.5726, 1.2529)	0.7185 (0.5365, 0.9623)	0.7598 (0.5825, 0.9909)	0.0279	
Never	reference	1.1490 (0.9003, 1.4666)	0.9008 (0.7160, 1.1334)	1.0211 (0.8582, 1.2150)	0.8159	
Now	reference	1.0729 (0.7259, 1.5857)	0.8798 (0.6497, 1.1914)	0.8258 (0.6051, 1.1271)	0.1944	
**Alcohol status**	reference					0.5099
Moderate	reference	1.1342 (0.6729, 1.9120)	0.8288 (0.5707, 1.2034)	0.9212 (0.6273, 1.3529)	0.451	
Never	reference	1.4238 (0.9073, 2.2343)	0.7213 (0.4836, 1.0759)	0.9376 (0.6311, 1.3930)	0.3723	
Mild	reference	0.7737 (0.5745, 1.0420)	0.8190 (0.6355, 1.0553)	0.8660 (0.6887, 1.0889)	0.2337	
Heavy	reference	1.2726 (0.8099, 1.9996)	0.7429 (0.5351, 1.0314)	0.8128 (0.5864, 1.1266)	0.062	
Former	reference	0.9259 (0.5459, 1.5703)	1.0672 (0.7563, 1.5058)	0.8802 (0.6485, 1.1946)	0.5951	
**DM**	reference					0.9137
No	reference	1.0434 (0.8495, 1.2817)	0.8302 (0.7146, 0.9646)	0.8756 (0.7554, 1.0150)	0.0226	
Yes	reference	0.9410 (0.6111, 1.4491)	0.8151 (0.5907, 1.1246)	0.8799 (0.6312, 1.2266)	0.3424	
**Hypertension**	reference					0.3535
Yes	reference	1.1894 (0.9165, 1.5436)	0.9230 (0.7429, 1.1469)	1.0088 (0.8176, 1.2447)	0.7778	
No	reference	0.9153 (0.6943, 1.2067)	0.7566 (0.6181, 0.9262)	0.7859 (0.6422, 0.9618)	0.0065	
**Research cycles**	reference					0.9175
1999–2000	reference	1.1687 (0.6531, 2.0915)	0.6425 (0.3658, 1.1287)	0.8413 (0.5426, 1.3044)	0.1973	
2001–2002	reference	0.9795 (0.5378, 1.7839)	0.7816 (0.4628, 1.3200)	0.8658 (0.6439, 1.1642)	0.2264	
2003–2004	reference	1.4175 (0.7496, 2.6804)	0.9174 (0.4718, 1.7840)	0.9119 (0.4845, 1.7162)	0.6332	
2009–2010	reference	1.1992 (0.7426, 1.9364)	0.8200 (0.6546, 1.0272)	0.9075 (0.6658, 1.2369)	0.3102	
2011–2012	reference	0.9188 (0.5512, 1.5317)	0.9887 (0.7124, 1.3723)	0.9295 (0.6716, 1.2865)	0.68	
2013–2014	reference	0.7679 (0.5219, 1.1300)	0.8492 (0.6539, 1.1028)	0.8851 (0.6928, 1.1309)	0.2363	
Each subgroup analysis was adjusted for age, gender, race, PIR, education, BMI, research cycles, smoking status, alcohol status, DM, and hypertension, except for the subgroup variable. Med, medium dietary live microbe intake; OR, odds ratio; CI, confidence interval; DM, diabetes mellitus; BMI, body mass index; PIR, poverty income ratio.
